# Geographical variations and contextual effects on age of initiation of sexual intercourse among women in Nigeria: a multilevel and spatial analysis

**DOI:** 10.1186/1476-072X-7-27

**Published:** 2008-05-30

**Authors:** Olalekan A Uthman

**Affiliations:** 1 Center for Evidence-Based Global Health, Save the Youth Initiative, Ilorin, Kwara state, Nigeria

## Abstract

**Background:**

The age of initiation of sexual intercourse is an increasingly important issue to study given that sexually active young women are at risk of multiple outcomes including early pregnancies, vesico-vaginal fistula, and sexually transmitted infections. Much research has focused on the demographic, familial, and social factors associated with sexual initiation and reasons adolescents begin having consensual intercourse. Less is known, however, about the geographical and contextual factors associated with age of initiation of sexual intercourse. Therefore, the purpose of this study was to examine the extent of regional and state disparities in age of initiation of sexual intercourse and to examine individual- and community-level predictors of early sexual debut.

**Methods:**

Multilevel logistic regression models were applied to data on 5531 ever or currently married women who had participated in 2003 Nigeria Demographic and Health Survey. Coital debut at 15 years or younger was used to define early sexual debut. Exploratory spatial data analysis methods were used to study geographic variation in age at first sexual intercourse.

**Results:**

The median age at first sexual intercourse for all women included in the study was 15 years (range; 14 – 19). North West and North East had the highest proportion of women who had reported early sexual debut (61% – 78%). The spatial distribution of age of initiation of sexual intercourse was nonrandom and clustered with a Moran's I = 0.635 (p = .001). There was significant positive spatial relationship between median age of marriage and spatial lag of median age of sexual debut (Bivariate Moran's I = 0.646, (p = .001). After adjusting for both individual-level and contextual factors, the probability of starting sex at an earlier age was associated with respondents' current age, education attainment, ethnicity, region, and community median age of marriage.

**Conclusion:**

The study found that individual-level and community contextual characteristics were independently associated with early sexual debut, suggesting that interventions to reduce adolescent high-risk sexual behaviour should focus on high-risk places as well as high-risk groups of people.

## Background

The age of initiation of sexual intercourse is an increasingly important issue to study given that sexually active young women are at risk of multiple outcomes including early pregnancies, vesico-vaginal fistula, and sexually transmitted infections. To date, most studies on the effects of early initiation of sexual intercourse among women have focused on those who had their sexual debut before marriage. Bruce et al[1] found that 87 out of 100 girls in Nigeria who had sex in the previous week were married. In the northern regions of Nigeria, where child marriage is common, virtually all sexual activity among girls occurs within the context of marriage. In Nigeria, where women are more susceptible to HIV, child marriage and early sexual intercourse may be significant risk factors for adolescent girls [2]. Some 3.9 percent of Nigeria's population is infected with HIV [3]. Girls aged 15–24 are about twice as likely as boys the same age to be infected [4]. Teenage girls are also more susceptible than older women to sexually transmitted infections (STI)[4].

Much research has focused on the demographic, familial, and social factors associated with sexual initiation and reasons adolescents begin having consensual intercourse [5-7]. Less is known, however, about the geographical and contextual factors associated with age of initiation of sexual intercourse. In the present investigation, more appropriate and recent multi-level modelling techniques were used to examine the association between age of initiation of sexual intercourse and contextual factors. The analysis of geographic variation in age of sexual initiation is important. First, such a focus is consistent with the Nigerian national health initiative, which aims to reduce and ultimately eliminate health inequalities among gender, racial/ethnic, socioeconomic, and geographic groups. In addition, a geographic analysis should help identify states and regions of the country that have a relatively high proportion of women with high risk sexual behaviour, which may in turn lead to the development and implementation of more effective geographically differentiated intervention programs. Exploratory spatial data analysis (ESDA)[8] methods were used to study geographic variations in age of initiation of sexual intercourse in Nigeria. ESDA is an extension of exploratory data analysis (EDA) that focuses on detecting spatial patterns in data and the generation of hypotheses based on the spatial patterns in the data.

Therefore, the purpose of this study was (1) to estimate prevalence of early sexual debut among ever or currently married women in six geographic regions (37 states) of Nigeria using a large, nationally representative sample, (2) to examine the extent of regional and state disparities in age of initiation of sexual intercourse, (3) to identify individual and contextual predictors of early sexual debut, and (4) to study the association between age of marriage and age of initiation of sexual intercourse.

## Methods

### Setting

Nigeria is located in western Africa on the Gulf of Guinea and has a total area of 923,768 kilometer squared (km^2^), making it the world's 32nd-largest country (after Tanzania). Nigeria is the most populous country in Africa. The United Nations estimates that the population in 2004 was at 131,530,000, with the population distributed as 48.3% urban and 51.7% rural and population density at 139 people per km^2^. Nigeria has more than 250 ethnic groups, with varying languages and customs, creating a country of rich ethnic diversity. The largest ethnic groups are the Fulani/Hausa, Yoruba, Igbo, accounting for 68% of population, while the Edo, Ijaw, Kanuri, Ibibio, Ebira Nupe and Tiv comprise 27%; other minorities make up the remaining 5%. The middle belt of Nigeria is known for its diversity of ethnic groups, including the Pyem, Goemai, and Kofyar.

One third of Nigeria's total population are youth between the ages of 10 and 24[9]. By 2025, the number of Nigerian youth will exceeds 57 million [9]. Lack of sexual health information and services places these young people at risk for pregnancy, abortion, sexually transmitted infections (STI), and HIV/AIDS [9]. In addition, early marriage and childbearing limit youth's educational and employment opportunities [9]. Among women ages 20 to 24, 19.8 percent reported having married by age 15, 39.6 percent by age 18, and 52.7 percent by age 20. In 1999, Nigeria's adolescent fertility rate was 111 births per 1,000 women ages 15 to 19, and Nigerian women averaged more than five births during their lifetime[9].

### Study design

Cross-sectional and population-based study using data from the 2003 Nigeria Demographic and Health survey (NDHS).

### Sampling technique

Methods used in the NDHS have been published elsewhere [10]. Briefly, the survey used a two-stage cluster sampling technique. The country was stratified into 36 states and the Federal Capital Territory (FCT) of Abuja. Each domain is made up of enumeration areas (EAs) established by a general population and housing census in 1991. The sampling frame was a list of all EAs (clusters). Within each domain, a two-stage sample was selected. The first stage involved selecting 466 clusters (primary sampling units) with a probability proportional to the size, the size being the number of households in the cluster. The second stage involved the systematic sampling of households from the selected clusters. A nationally representative probability sample of 7864 households was then selected from the clusters, in which all women aged 15 to 49 years were eligible to be interviewed.

### Data collection

Data collection procedures have been published elsewhere [10]. Briefly, data were collected by visiting households and conducting face-to-face interviews to obtain information on demographic characteristics, wealth, anthropometry, female genital cutting, HIV knowledge, and sexual behaviour between March and August 2003.

### Ethical consideration

This study is based on an analysis of existing survey data with all identifier information removed. The survey was approved by the Ethics Committee of the ORC Macro at Calverton in the USA and by the National Ethics Committee in the Ministry of Health in Nigeria. All study participants gave informed consent before participation and all information was collected confidentially.

### Variables

#### Outcome variable

For the present study, 5531 ever or currently married women, all of whom who have had at least one episode of sexual intercourse in their lifetime, were drawn from the overall sample of 7620 women to examine factors associated with early sexual initiation. Thus, if a woman has never had sexual intercourse in her life (e.g., a "virgin") and never married, she was not included in the analysis. Coital debut at 15 years or younger (Yes or No) was used to define early sexual debut. The cut-off point for age at coital debut was based on the median values of Nigeria women included in the study.

#### Explanatory variables

Four variables characterising individual-level features shown to affect age of initiation at first sexual intercourse were included in this study. The variables were age, educational attainment, ethnicity and religion affiliation. There were five community-level variables. These include: the degree of ethnic diversity in a community, the community urbanization measure (urban residence) and regional dummies (region). The other contextual variables include community median age of marriage and community median spousal age gap. The full definition and coding for each explanatory variable is given in Table 1.

**Table 1:** Variables, measures, and coding

***Variable***	***Measure***
***Dependent variable***	
Early sexual debut	Coital debut at 15 years or younger (yes = 1, no = 0)
***Individual characteristics***	
Age	Completed years [1) 15–24, 2) 25–34, 3) 35+]
Education	Highest level of education [1) No education, 2) primary, 3) secondary or higher]
Ethnicity	Ethnicity [1) Hausa or Fulani, 2) Igbo, 3) Yoruba, 4) Others]
Religion	Religion affiliation [1) Islam, 2) Catholic, Protestant, Other Christian, 3) traditionalist, others]
***Community characteristics***	
Type of residence	Cluster classification [1) rural or remote, 2) urban]
Community ethnic diversity	Number of ethnic group in a community, represents the likelihood that two persons, chosen at random from the same area, belong to different ethnic group.
Community median age of marriage	Median age of women at first marriage in community
Community median spousal age gap	Median spousal age gap in community
Region	The six geopolitical regions [1) north-central, 2) north-east, 3) north-west, 4) south-east, 5) south-south, 6) south-west]

### Statistical analyses

#### Descriptive and bivariate association

The descriptive statistics show the distribution of respondents by the key variables. Values were expressed as absolute numbers (percentages) and mean (standard error) for categorical and continuous variables respectively. Contingency tables were analyzed using Pearson's chi-squared test. For continuous variable, a Mann-Whitney test was used to examine the association between early sexual debut and each explanatory variable. All cases in the DHS data were given weights to adjust for differences in probability of selection and to adjust for non-response in order to produce the proper representation. Individual weights were used for descriptive statistics in this study, using Stata 10 for Windows [11].

#### Multilevel modelling

Given the hierarchical structure of the sample and the binary outcome, a logistic multilevel modeling approach was adopted [12]. A three-level model with a binary response (*y*, reported as having started sexual intercourse at an early age) for women *i *living in community *j *in state *k *of the form:

(1) *π*_*ijk*_: *y*_*ijk*_   ~Bemoulli(1, *π*_*ijk*_)

The probability was related to a set of categorical predictors, *X*; and a random effect for each level, by a logit-link function as

(2) logit(*π*_*ijk*_) = log [*π*_*ijk*_/(1-*π*_*ijk*_)] = *β*_0 _+ *βX*_*ijk *_+ *u*_0*jk *_+ *v*_0*k*_

The linear predictor on the right-hand side of the equation consisted of a fixed part (*β*_0 _+ *βX*_*ijk*_) estimating the conditional coefficients for the explanatory, and two random intercepts attributable to communities (*u*_0*jk*_) and states (*v*_0*k*_) with each assumed to have an independent and identical distribution and variance estimated at each level.

The analysis was done in three steps. In Model 1 (empty model), no explanatory variable was included. In Model 2, only individual-level factors were included. In Model 3, community contextual factors were added to Model 2. The results of fixed effects (measures of association) were shown as odds ratios (ORs) with 95% confidence intervals (CIs). The results of random effects (measures of variation) were presented as variance partition coefficient and percentage change in variance.

The MLwiN software, version 2.0.2 [13], was used for the analyses. Parameters were estimated using the Markov Chain Monte Carlo (MCMC) procedure. The default settings in MLwiN were used for the analyses, i.e., chains of length 5000 after a burn-in of 500. The Deviance Information Criterion (DIC) was used as a measure of how well our different models fitted the data. A lower value on DIC indicates a better fit of the model [14,15].

#### Exploratory spatial data analysis (ESDA)

The spatial distribution of the rate of early sexual initiation was first visualized and spatially smoothed prevalence was obtained by means of spatial empirical Bayes estimation[8]. The spatial pattern of prevalence of early sexual debut was analyzed using Univariate LISA Moran Scatter plot[16]. The bivariate spatial relationship between age of sexual initiation and age of marriage was studied using bivariate Moran's I[17]. The bivariate LISA (BiLISA) Moran's I "gives an indication of the degree of linear association (positive or negative) between the value for one variable at a given location *I *and the average of another variable at neighboring locations." Similar to LISA, BiLISA suggests two classes of positive spatial correlation, or spatial clusters (High-High and Low-Low), and two classes of negative spatial correlation, or spatial outliers (High-Low and Low-High). Inference for Moran's I was based on a permutation approach, in which a reference distribution is calculated for spatially random layouts with the same data as observed. The randomization uses an algorithm to generate spatially random simulated data sets as outlined by Anselin[18]. 999 random permutations were used in constructing the reference distribution. ESDA is implemented through the *GeoDa *software[19]. GeoDa provides a very user-friendly environment to implement ESDA methods and is freely downloadable.

## Results

### Sample characteristics and bivariate associations

The weighted descriptive statistics are presented in Table 2, 5531 ever or currently married women nested within 177 communities nested within 37 states were analyzed in this study. Among women included in the study, nearly half (48%) reported first sex by age 15. Most of the respondents reside in the rural areas (69%), and were Muslim (60%). The results of bivariate associations with early sexual debut are presented in Table 3. Compare to Christian women, Muslim women were more likely to have started sex intercourse at an earlier age (62% versus 38%, p = .001). Counterintuitively, urban women were less likely to have reported early sexual debut (39% versus 54%; p = .001). Compared to Hausa women, Yoruba (14% vs. 71%) and Igbo women (23% vs. 71%) were less likely to have started sex early. In addition, compared to their counterparts in the southern part of the country, women from northern regions were more likely to have started sex at an early age.

**Table 2:** Socio-demographic characteristics of ever or currently married women, Nigeria 2003*

***Measure/variable***	***Weighted sample (percentage)***
***Dependent variable***	
Early sexual debut (15 years or less)	
Yes	2755 (48.4)
No	2937 (51.6)
***Individual characteristics***	
Age (completed years)	
15 – 24	1523 (26.8)
25 – 34	2106 (37.0)
35+	2063 (36.2)
Education	
No education	3044 (53.5)
Primary	1266 (22.2)
Secondary or higher	1381 (24.3)
Ethnicity	
Hausa/Fulani	2387 (41.9)
Igbo	582 (10.2)
Yoruba	535 (9.4)
Others	2168 (38.1)
Religion	
Christian	2156 (37.9)
Islam	3444 (60.5)
Others	89 (1.6)
***Community characteristics***	
Type of residence	
Rural	3907 (68.6)
Urban	1785 (31.4)
Community ethnic diversity^†^, Mean (SE)	3.7 (0.09)
Community median age of marriage, Mean (SE)	16.2 (0.11)
Community median spousal age gap, Mean (SE)	9.7 (0.08)
Geopolitical region	
North-central	807 (14.2)
North-east	1197 (21.2)
North-west	1949 (34.2)
South-east	396 (7.0)
South-south	764 (13.4)
South-west	579 (10.2)

Abbreviation – SE – Standard Error

^†^Number of ethnic group in a community, represents the likelihood that two persons, chosen at random from the same area, belong to different ethnic group

*Data source – 2003 Nigerian Demographic and Survey. Sample size – 5531

**Table 3:** Bivariate association between early sexual debut and selected individual compositional and community contextual characteristics of ever or currently married Nigerian women, 2003*

	***Early sexual debut***		
	No [%(n)]	Yes [%(n)]	p-value

***Individual characteristics***			
Age (completed years)			.001
15 – 24	46.6 (640)	53.4 (735)	
25 – 34	56.0 (1159)	44.0 (910)	
35+	50.6 (1057)	49.4 (1030)	
Education			.001
No education	35.3 (1013)	64.7 (1856)	
Primary	57.1 (724)	42.9 (543)	
Secondary or higher	80.2 (1119)	19.8 (276)	
Ethnicity			.001
Hausa/Fulani	29.2 (606)	70.8 (1467)	
Igbo	76.7 (578)	23.3 (176)	
Yoruba	85.5 (541)	14.5 (92)	
Others	54.7 (1122)	45.3 (930)	
Religion			.001
Christian	69.9 (1593)	38.1 (686)	
Islam	38.4 (1202)	61.6 (1932)	
Others	52.6 (60)	47.4 (54)	
***Community characteristics***			.001
Type of residence			
Urban	60.7 (1247)	39.3 (807)	
Rural	46.3 (1609)	53.7 (1868)	
Community ethnic diversity^†^, Mean (SD)	3.5 (2.6)	3.9 (2.5)	.001
Community median age of marriage, Mean (SD)	17.5 (2.5)	15.4 (1.9)	.001
Community median spousal age gap, Mean (SD)	9.3 (2.3)	10.2 (1.8)	.001
Geopolitical region			.001
North-central	60.4 (553)	39.6 (363)	
North-east	38.1 (523)	61.9 (750)	
North-west	32.0 (523)	67.8 (1101)	
South-east	78.5 (442)	21.5 (121)	
South-south	58.8 (314)	41.2 (220)	
South-west	82.4 (562)	17.6 (120)	

Chi-squared test was used for categorical data, and Mann-Whitney test continuous data for the bivariate analysis. For continuous variables, results are shown as mean and standard deviation.

^†^Number of ethnic group in a community, represents the likelihood that two persons, chosen at random from the same area, belong to different ethnic group

Data source – 2003 Nigeria Demographic and Survey. Sample size – 5531

### Exploratory spatial data analysis

**Figure 1:**
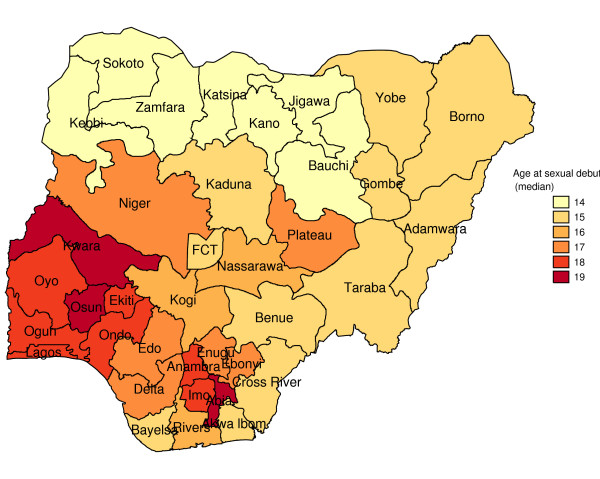
Map showing median age of sexual initiation by states.

**Figure 2:**
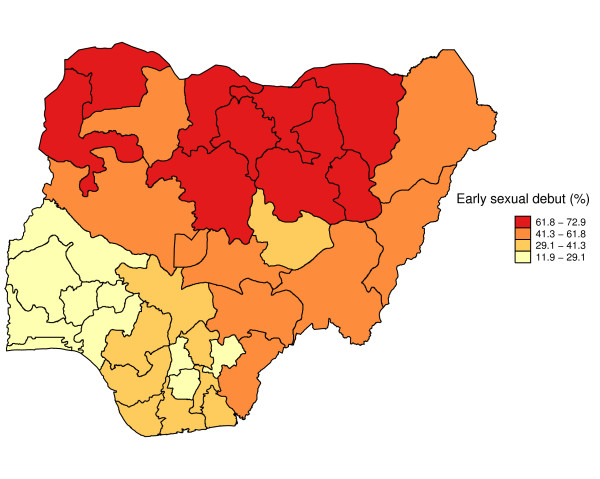
Map showing the spatial empirical bayes smoothed proportions of women who reported early sexual initiation at state level.

**Figure 3:**
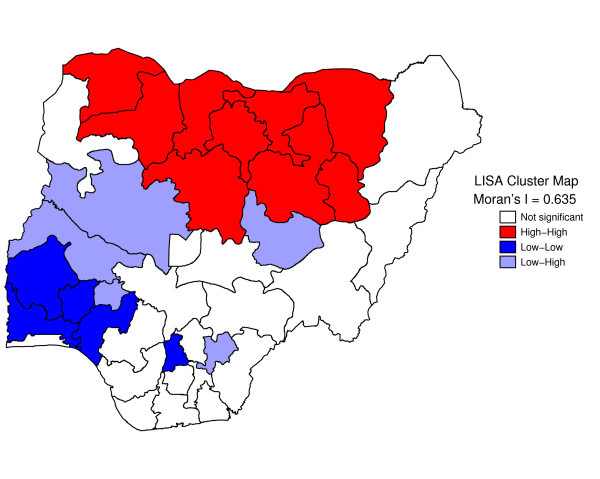
Local Indicator of Spatial Association (LISA) cluster map for Nigeria women age of initiation sexual intercourse.

**Figure 4:**
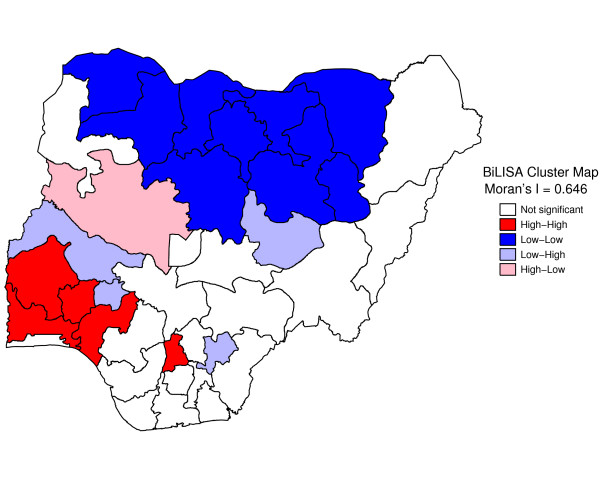
Bivariate LISA (BiLISA) cluster map for community median age of marriage and spatial lag of community median age of sex initiation in Nigeria, 2003.

Figure 1 shows geographic variation in state-specific median age of sexual initiation. The median age of sexual debut was lowest in North West (14 years) and highest in Kwara, Osun, and Abia states (19 years). Figure 2 presents a state map of the smoothed proportion of women who had reported early sexual debut. The smoothed proportions present clearer patterns and show clearly, where the problem was most severe. The spatial empirical bayes "smooth" estimates technique was able to deal with spatial heterogeneity. The estimation technique guarantees that estimates of neighboring states are more alike than estimates of states that are far away. North West and North East had the highest proportion of women who had reported early sexual debut (61% to 78%). The proportion of women who had reported early sexual intercourse was lowest in South West, South South, South East, and Kwara State (8% to 25%). The prevalence of early sexual debut was checked for spatial patterns by use of a Moran scatter plot. The results of this plot show statistically significant autocorrelation (Moran's I = 0.635, p = .0001) (Figure 3). In addition, the bivariate Local LISA Moran's I statistics for spatial correlation between median age of marriage and spatial lag of median age of sex initiation is 0.646, (p = .001), indicating a significant positive spatial relationship between median age of marriage and median age of initiation of sexual intercourse. As expected, in the northern regions there is significant correlation between low median age of marriage and low median age of initiation of sexual intercourse neighbours (Figure 4).

### Multilevel analyses

The result of the random effects model is shown in Table 4 (Empty Model 1). There is significant variation in the log odds of early sex debut across the communities (*τ *= 0.289, *p *= .001) and states (*τ *= 1.058, *p *= .001). According to the intra-state correlation coefficient implied by the estimated intercept component variance, 22.8% and 6.0% of the variance in the age of initiation of sexual intercourse could be attributed to state-level and community-level variables respectively. This variation remained significant, even after controlling for individual-level factors (Model 2). However, the unexplained variations that remained after controlling for both individual and community-level characteristics were not significant (Model 3). As judged by the proportional change in variance, 79% and 95% of the variance in the log odds of early sexual debut variance across states was explained by individual compositional factors (Model 2) and both individual compositional and contextual factors (Model 3) respectively. 63% and 92% of the variance in the log odds of early sexual debut variance across communities was explained by individual compositional factors (Model 2) and both individual compositional and contextual factors (Model 3) respectively. In addition, the deviance information criterion (DIC) – was significant to reveal that the individual compositional factors (Model 2) and individual compositional and contextual variables (Model 3) increased the multivariable multilevel model's ability to explain variation in the log odds of early sexual debut, as indicated by lower DIC.

**Table 4:** Individual compositional and community contextual factors associated with early sexual debut among ever or currently marriage Nigeria women identified by multivariable multilevel logistic regressions^‡^

	***Empty model***	***Individual variables***	***Individual and community variables***
	
	**Model 1**	**Model 2**	**Model 3**
	
	**OR (95% CI)**	**OR (95% CI)**	**OR (95% CI)**
**Fixed effects**			
***Individual characteristics***			
Age (completed years)			
15 – 24		1	1
25 – 34		1.02 (0.87, 1.21)	1.05 (0.89, 1.24)
35+		1.19 (1.02, 1.41)*	1.24 (1.05, 1.47)**
Education			
No education		1	1
Primary		0.69 (0.59, 0.80)***	0.74 (0.62, 0.88)***
Secondary or higher		0.26 (0.22, 0.70)***	0.30 (0.25, 0.36)***
Ethnicity			
Hausa/Fulani		1	1
Igbo		0.35 (0.23, 0.52)***	0.53 (0.34, 0.80)**
Yoruba		0.22 (0.14, 0.33)***	0.32 (0.21, 0.48)***
Others		0.56 (0.45, 0.70)***	0.63 (0.51, 0.78)***
Religion			
Islam		1	1
Christian		0.77 (0.61, 0.96)*	0.81 (0.64, 1.02)
Others		1.02 (0.65, 1.59)	0.92 (0.60, 1.45)
***Community characteristics***			
Type of residence			
Urban			1.10 (0.95, 1.25)
Rural			1
Community ethnic diversity^†^			0.98 (0.95, 1.01)
Community median age of marriage			0.77 (0.77, 0.81)***
Community median spousal age gap			1.01 (0.98, 1.05)
Geopolitical region			
North-central			0.44 (0.31, 0.64)***
North-east			0.40 (0.24, 0.66)***
North-west			0.48 (0.30, 0.77)**
South-east			0.43 (0.24, 0.75)**
South-south			1
South-west			0.57 (0.34, 0.96)*
Constant			
**Random effects**			
State-level			
Variance (SE)	1.058 (0.289)***	0.220 (0.084)**	0.057 (0.03)
VPC (%)	22.8	6.1	1.7
Explained variation (%)	Reference	79.2	94.6
Community-level			
Variance (SE)	0.280 (0.057)***	0.104 (0.039)**	0.021 (0.017)
VPC (%)	6.0	2.9	0.6
Explained variation (%)	Reference	62.8	92.5
**Model fit statistics**			
DIC	6543	6255	6219
Sample size			
State-level	36	36	36
Community-level	177	177	177
Individual-level	5531	5508	5508

Abbreviations: OR – Odds ratio, CI – Confidence interval, VPC – Variance Partition Coefficient, SE – Standard error, DIC – Deviance information criterion

†Number of ethnic group in a community, represents the likelihood that two persons, chosen at random from the same area, belong to different ethnic group

*p < .05, **p < .01, and ***p < .001

^‡^Data source: 2003 Nigerian Demographic and Health Survey

The results of fitting the model including individual-level variables appear in Table 4 (Model 2). The log odds of early sexual debut increased with increasing age and decreased with increasing level of education. Christian women were less likely to have started sex at early age. Compared to their counterparts that are Hausa or Fulani, Igbo and Yoruba women were 65% and 88% less likely to have reported early initiation of sexual intercourse respectively. The effect of the inclusion of contextual factors is shown in Table 4 (Model 3). Inclusion of the community-level variables had minimal effect on the contribution of individual-level variables to the likelihood of starting sex at an early age. After individual compositional and contextual factors were added, regional dummies and community median age of marriage were statistically significant with early sexual debut. Independent of other factors, higher community median age of marriage was significantly associated with a decreased likelihood of started sex at early age, with 26% lower likelihood with every one-year increase in the community median age of marriage.

## Discussion

### Main findings

Using an explicit multilevel analytic framework and exploratory spatial data analysis, the study has shown that both individual composition and community contextual characteristics are important predictors of women's age at first sexual intercourse, and demonstrates geographic variation in rates of early initiation of sex in Nigeria. After adjusting for both individual-level and contextual factors, the probability of starting sex at an earlier age was associated with respondents' current age, educational attainment, ethnicity, region, and community median age of marriage. This paper has offered an alternative to more traditional ways of thinking about the distribution of sexual behaviour and sexual risk at the population level. The potential of multilevel modelling and exploratory spatial data analysis for exploring population contextual influences on individual sexual behaviour is clearly established.

Nigeria is made up of six major geopolitical regions. It is ethnically and religiously diverse and economic development and education levels vary widely across the country[20]. Not unexpectedly, this study found that Nigerian women's age of initiation of sexual intercourse varies widely by region. Specifically, traditional and cultural beliefs that seem to encourage early marriage and lead to early initiation of sexual intercourse may be principal reasons for the observed geographical disparities. The study found that women living in neighborhoods with low median age of marriage had increase odds of reporting early initiation of sexual intercourse than those in neighborhoods with higher median age of marriage. This is consistent with the preponderance of evidence suggesting that Northern regions of Nigeria have some of the highest rates of early marriage in the world.

### Study strengths and Limitations

There are a number of caveats to be considered when interpreting these results. The cross-sectional nature of the data limits ability to draw casual inferences. For this study, regions were administratively defined. The main drawback of this is that these boundaries may not match residents' perceptions and lived experiences of their "regional" boundaries. Another important limitation is validity constraints of self-reported sexual activity. By definition, self-reports of initiation of sexual activity cannot be externally validated and studies have revealed considerable inconsistencies in individual's self-reported sexual activity [21,22]. According to a study of eight African Demographic and Health Surveys by Meekers [22], recall errors and misinterpretation of questions about first sexual intercourse may be responsible for misreporting of sexual behaviour.

Instead of reporting the age at which they first ever had sexual intercourse, some women report the age at which they first had sex with either their first or current husband [22]. Lauritsen et al[21] found that adolescents may under report sexual activity, depending on how recently that activity took place relative to the timing of the interview. Recalling age at first intercourse has been found to be significantly related to race, gender and family structure [21]. In addition to standard limitations such as self-reported data and recall bias, the study was limited in that an existing data set used was not designed specifically to answer the research questions. Finally, spatial clustering methods are exploratory tools that help researchers and policy makers make sense of complex geographic patterns. Once a cluster is identified, only detailed epidemiological investigation can determine if the cluster is a "random" event. Therefore, it is essential that statistical significant clusters be examined in more detail. Despite these limitations, the study strengths are significant. It relies on a large, population-based study with national coverage. Individual compositional and community contextual characteristic accounted for 95% and 92% of the state and community disparities in early sexual debut, illustrating that the models were effective in predicting the risk of early sexual debut, and inclusion of contextual factors eliminated the significance of the state- and community-level variance (**Model 3**).

### Policy implications

The study findings have some important and relevant policy messages. The findings demonstrate that both individual compositional and community contextual factors are key factors that predict adolescent's risk of becoming sexually active at an earlier age. Better identifying and understanding the interplay between these individual-level characteristics and a variety of social and cultural contexts can provide valuable information for planners who determine the allocation of scarce resources. In addition, understanding of regional differences may aid in the identification of regions that may need to be particularly targeted with education and prevention programs. Multifaceted geographically differentiated intervention may represent a potentially effective approach for addressing issues related to adolescent sexuality. Program components could include: youth development, media campaigns, educational interventions, male involvement initiatives and legislative reform efforts.

## Conclusion

This first known national multilevel and spatial study of age at first sexual intercourse found strong evidence that women's odds of starting sexual intercourse early was significantly associated with respondents' current age, education attainment, religion affiliation, ethnicity, geopolitical region, and community median age of marriage. The study also shows that substantial geographical variations in rates of early initiation of sex exist within Nigeria. The findings suggest that multifaceted geographically differentiated interventions may represent a potentially effective approach for addressing issues related to adolescent sexuality. Scholars trying to understand variation in individual high risk sexual behaviour should pay attention to the characteristics of both individuals and places of residence.

## Competing interests

The author declares that they have no competing interests.

## Authors' contributions

OAU conceived the study, extracted the data, did the analyses and interpretation, and wrote the first and final draft of the manuscript.
